# Exploring oral health indicators, oral health-related quality of life and nutritional aspects in 23 medicated patients from a short-term psychiatric ward

**DOI:** 10.3389/fpubh.2023.1083256

**Published:** 2023-04-12

**Authors:** Julie Frigaard, Håvard Hynne, Karoline Randsborg, Tonje Mellin-Olsen, Alix Young, Morten Rykke, Preet Bano Singh, Lene Hystad Hove, Anne Kristine Hofgaard, Janicke Liaaen Jensen

**Affiliations:** ^1^Department of Oral Surgery and Oral Medicine, Institute of Clinical Dentistry, University of Oslo, Oslo, Norway; ^2^Clinical Nutrition Unit, Lovisenberg Diaconal Hospital, Oslo, Norway; ^3^Department of Cariology and Gerodontology, Institute of Clinical Dentistry, University of Oslo, Oslo, Norway; ^4^Department of Psychiatry, Lovisenberg Diaconal Hospital, Oslo, Norway

**Keywords:** xerostomia, hyposalivation, diet, dental health, meal pattern, saliva, nutritional, psychiatry

## Abstract

**Background:**

Patients suffering from psychiatric disorders face many difficulties due to their condition, medications and lifestyle. Oral health and nutrition may be affected, further complicating their lives. Our aim was to provide in-depth information on oral health and nutritional factors in a small group of patients in short-term psychiatric ward.

**Methods:**

Twenty-three patients (mean age 36, average medications five) were recruited during short-term hospitalization in a psychiatric ward. Inclusion criteria: anxiety, psychosis and/or depression, and use of at least one antidepressant or anxiolytic/antipsychotic drug with xerostomia as a known side effect. Subjective oral dryness was evaluated using the Shortened Xerostomia Inventory (SXI). Oral examination included Clinical Oral Dryness Score (CODS), secretion of unstimulated (UWS) and stimulated whole saliva (SWS), and evaluation of dental, gingival, and periodontal status. Self-reported complaints of oral disorders were recorded. The Oral Health Impact Profile-14 (OHIP-14) was used to explore oral health-related quality of life. Nutritional status was assessed using the Patient-Generated Subjective Global Assessment Short Form (PG-SGA-SF), and diet quality was assessed using the Mediterranean diet score (KIDMED).

**Results:**

Compared to healthy controls, the patients had significantly higher SXI scores and CODS, and SWS secretion was lower. Complaints of dysgeusia and halitosis were significantly more frequent among patients. Gingivitis was more common in patients. OHIP-14 scores were much higher in the patients, and they reported significantly poorer oral and general health. Most patients lacked a regular meal pattern. Very low diet quality was observed in five patients, while improvements were needed in twelve. “*Dry mouth*” and “*No appetite, just did not feel like eating*” were the most common symptoms preventing patients from eating enough. The PG-SGA-SF symptoms component score showed a strong negative correlation with self-reported oral health, and a strong positive correlation with OHIP-14.

**Conclusion:**

This relatively small group of patients in short-term psychiatric ward had both reduced oral health and poor oral health-related quality of life. Furthermore, their nutritional intake was affected by their oral health problems. Although larger groups need to be studied, these findings indicate that oral health and nutrition should be evaluated and adjusted in these patients to improve their overall care.

## 1. Introduction

Mental and oral health are shown to be closely related (1). Many studies have shown that psychiatric patients with severe disease have reduced oral health (2–8). Patients with milder psychiatric diseases such as depression and anxiety also have reduced oral health, but to a lesser degree (9–11). The presence of a psychiatric diagnosis has also been shown to negatively affect dental hygiene routines and dental visits (7). Additionally, psychiatric disorders may have an impact on the financial situation of the patient due to unemployment and sick leave, hence providing a reduced dental visit pattern (12).

Saliva comprises a key factor in the first-line of defense against microbial damage, and poor saliva secretion may lead to an accumulation of microorganisms in the oral cavity (13). Dry mouth is a known side effect of many of the drugs used in the treatment of psychiatric patients, and reduced saliva secretion is therefore a well-established risk factor for reduced oral health in psychiatric patients.

Oral health includes several aspects concerning the mouth, such as the ability to speak, smile, taste, smell, chew and swallow (14). Good oral health is characterized by the absence of pain, discomfort and disease in the oral cavity (14). In this paper, dental health refers to teeth and their surrounding tissues such as gingiva and alveolar bone and is a part of oral health, although results from the clinical examinations are described independently for structural purposes. Poor oral health may interfere with nutrition by restricting or increasing intake of certain foods. In turn, this may influence body weight and general health, and thereby the overall quality of life of an individual (15, 16). Patients with reduced mental health, particularly severe mental illness, are at greater risk of developing oral health problems due to a specter of potential risk factors such as high consumption of sugary substances, poor oral hygiene, self-neglect, dehydration, vitamin deficiency and concomitant misuse of substances such as tobacco and alcohol (5, 9, 11). The lack of routine oral health examination and treatment in mental health care facilities, has been reported as a problem both by persons living with mental illnesses and by psychiatrists themselves (12).

The aim of the present paper was to provide in-depth information on oral health and nutritional factors in a small group of patients in short-term psychiatric ward.

## 2. Materials and methods

### 2.1. Study participants

This explorative cross-sectional study was a collaboration between the Department of Psychiatry and the Clinical Nutrition Unit at Lovisenberg Diaconal Hospital (LDH) and the Institute of Clinical Dentistry, Faculty of Dentistry, University of Oslo (Fac Dent). The study was performed from May 2021 to December 2021.

The patients were recruited from an elective short-term psychiatric ward (3–4 week stays), intended for patients with anxiety, depression, bipolar disorder, psychosis, personality disorders and eating disorders who can profit from planned inpatient stays. Referred patients were adults up to middle age, but mainly in the younger age groups. The treatment plan had a focus on group participation, physical activity, socialization, and education, taking into consideration that many of the patients had problems with loneliness, lack of belonging, lack of occupational affiliation, problems related to family, relational problems, minor substance abuse problems and economic problems. However, the patients’ global functioning was not seriously impaired. The patients were recruited during the Covid-19 pandemic. Infection control precautions led to reduction of beds and periods of closing the ward, affecting the number of eligible patients.

Upon admission to the ward, all patients fulfilling the inclusion criteria were asked to participate in the study. The inclusion criteria were patients diagnosed with anxiety, psychosis and/or depression, and the usage of one or more antipsychotic or antidepressant drug with xerostomia as a known side effect. The exclusion criteria were severe somatic illness, pronounced abuse problems, and body mass index (BMI) <16. Twenty-three patients agreed to participate in the study, although one did not complete the nutritional analysis. Twenty-three age and gender-matched healthy controls were recruited from Fac Dent. The participants in the control group had no previous complaints of oral dryness and did not use any medications that can affect salivary secretion rate.

The patients were recruited at the Department of Psychiatry at LDH. There they completed questionnaires about quality of life, with assistance if needed. Nutritional data collection by questionnaires and interviews were also carried out at the Department of Psychiatry. Orthopantomograms (OPGs) were taken at LDH, while all objective clinical oral investigations and subjective oral health-related evaluations were performed at the Dry Mouth Clinic at Fac Dent. Demographic data were collected during the clinical consultation at Fac Dent, while information concerning medical diagnoses and medications were provided by LDH. Data collected at Fac Dent was recorded electronically in the University Health Network Database (17). Finally, all sensitive data were transferred to and stored in the University of Oslo’s Services for sensitive data system (TSD).

The study protocol was approved by the Norwegian Regional Medical Ethical Committee (REK 2021/78549) and was performed in compliance with the tenets of the Declaration of Helsinki. Written informed consent was obtained from all participants prior to inclusion in the study. The data was de-identified prior to analysis.

### 2.2. Oral and dental evaluation

Xerostomia was evaluated using the Summated Xerostomia Inventory (SXI) questionnaire. SXI is a shortened version of the Xerostomia Inventory (18) and was used to determine the subjective severity of xerostomia. The SXI questionnaire consists of five statements with three alternative replies regarding the frequency of each statement. Each reply has a corresponding score: 1 = never, 2 = occasionally and 3 = often. The SXI sum score is 5–15, a maximum score indicating severe problems related to dry mouth. Furthermore, the investigators asked standardized questions concerning the participants overall oral and general health during the examination. These questions were graded on a scale from 0 to 4, where 0 indicated very bad and 4 indicated very good health conditions.

Unstimulated whole saliva (UWS) and chewing-stimulated whole saliva (SWS) were collected to determine saliva secretion rates. The participants were instructed to refrain from having anything in their mouth the last hour prior to saliva collections. First, UWS was collected for 5 min in pre-weighed plastic cups chilled on ice. The participants were instructed to allow all saliva produced during the 5 min to drip passively into the plastic cup. SWS was then collected in the same manner while the participants chewed on a paraffin wax tablet (Paraffin pellets, Ivoclar Vivadent, Shaen, Liechtenstein). The participants were instructed to chew on the paraffin tablet for approximately 30 s, and then swallow any saliva in the mouth before starting the 5 min of SWS collection into the plastic cup. The saliva samples were weighed, and saliva secretion rates were calculated for both UWS and SWS (g/mL = mL/min). Hyposalivation was defined as a salivary secretion rate of ≤0.1 ml/min for UWS and of ≤0.7 ml/min for SWS (19).

For an objective evaluation of oral dryness, the Clinical Oral Dryness Score (CODS) was used (20, 21). Ten clinical signs of oral dryness determine the CODS value, each positive feature scores 1 point with a subsequent total score ranging from 0 to 10. Higher scores indicate more severe oral dryness.

The dental status of all participants was recorded based on a thorough clinical examination, OPG and bite-wing radiographs. Standardized clinical photographs were taken of the teeth and tongue. Dental caries experience was evaluated using the DMF-index (total DMFT score range 0–28). The D_3_MFT score was recorded for all participants, being the sum of decayed teeth (D_3_T, i.e., at the level of dentine caries), filled teeth (FT), and missing teeth (MT). The presence of root-filled teeth and signs of tooth wear were recorded only at the individual level (yes/no), as was the presence of dental biofilm (plaque) and gingival inflammation. Periodontal status was recorded as either no periodontitis, localized periodontitis (attachment loss of ≥2 mm affecting two non-adjacent teeth, however, less than 30% of the dentition affected), and generalized periodontitis (attachment loss of ≥2 mm affecting more than 30% of the dentition). Twenty-one of the patients were also interviewed about their last dental visit, and their oral hygiene routines, including use and frequency of tooth brushing, toothpicks, and dental floss.

### 2.3. Oral and chemosensory disorders

Subjective oral complaints concerning oral conditions, such as previous oral candidiasis, bad breath (halitosis), bad taste (dysgeusia), and burning mouth sensation (BMS) were registered for all participants. Taste and smell functions were self-evaluated using a visual analogue scale (VAS) from 0 to 10, with 0 being *no function* and 10 being *very good function*.

The objective measurements of smell and taste functions were performed using 12 Sniffin’ Sticks (Burghart Messtechnik, Wedel, Germany), and 16 Taste Strips (Burghart Messtechnik, Wedel, Germany) with four taste qualities (sweet, sour, bitter, salty) in four different concentrations. For smell function, a smell identification test was performed. First, the participants received a multiple-choice card with four alternative smells and were instructed to read the card. Then, the odor pens were waved slowly approximately 2 cm from the participants’ nostrils for 3–4 s, and the participants were instructed to choose one of the alternatives. The responses were recorded on a protocol sheet, and the data was summarized for each patient. A normative classification was used to define the participants as either anosmic (0–5), hyposmic (6–9), or normosmic (10–12) (22).

The taste function test was performed by gently rubbing the taste strips on both sides of the anterior tip of the participants’ extended tongue, starting with the weakest concentration. A chart with names of the four taste qualities was placed in front of the participants during testing and they were instructed to give a forced choice answer. The taste qualities were presented in a random manner. The participants were allowed to rinse their mouth with water during the taste function testing. The responses were recorded on the protocol sheet, and the data was summarized for each patient. A normative classification was followed to distinguish between either augesic (score 0), hypogeusic (score 1–8) and normogeusic (score 9–16) participants (23).

The presence of oral candida was tested by rubbing a sterile cotton swab over the mucosa of one cheek and the anterior part of the tongue of the participants. Samples were inoculated on Sabourad’s dextrose agar plates, incubated for three to four days at 37°, and scored semi-quantitatively: 0 = no growth, 1 = minimal growth (1–9 colonies), 2 = moderate growth (10–29 colonies), 3 = severe growth (>30 colonies).

### 2.4. Oral health-related quality of life

All patients completed the Oral Health Impact Profile-14 (OHIP-14) questionnaire at LDH with access to assistance if needed. In addition, a set of questions regarding aspects of oral health’s impact on their social life were filled out at Fac Dent.

The OHIP-14 questionnaire is a short-form of the original OHIP-49 questionnaire (24), and provides a comprehensive measurement of self-reported dysfunction, discomfort and disability attributed to the oral conditions. The questionnaire consists of 14 questions, and the patients answer on a five-point scale representing the frequency of complaints for each question (0 = *never*, 1 = *hardly ever*, 2 = *occasionally*, 3 = *fairly often*, 4 = *very often*). The summated OHIP-14 score ranges from 0 to 56, where a high score indicates poor oral health-related quality of life (OHRQoL) (25). The OHIP-14 questionnaire is organized into seven dimensions addressing various aspects of oral health, two questions (Q) represent each dimension: functional limitations (Q1 + Q2), physical pain (Q3 + Q4), psychological discomfort (Q5 + Q6), physical disability (Q7 + Q8), psychological disability (Q9 + Q10), social disability (Q11 + Q12) handicap (Q13 + Q14) (25).

### 2.5. Nutrition risk screening

Twenty-two patients completed the Patient-Generated Subjective Global Assessment Short Form (PG-SGA-SF) questionnaire, assisted by a clinical nutritionist at LDH. The PG-SGA-SF consists of the patient-reported component of the original PG-SGA questionnaire and can be used separately as a malnutrition risk-screening tool (26, 27). It consists of four components: current and former body weight, emphasising recent weight loss (Box 1), current food and normal food intake (Box 2), symptoms that negatively influence food intake (Box 3), and activities and function (Box 4). The numerical scoring range is from 0 (no problems) to 36 (worst problems), where the four boxes have maximum scores of 5, 4, 24 and 3, respectively. Malnutrition risk was categorized as low (score 0–3), medium (score 4–8) or high (score ≥9).

### 2.6. Nutrition assessment

Food habits and preferences were assessed using a dietary interview including meal patterns, and intake of foods and drinks during the past month prior to hospital admittance. In addition, the patient’s responses to whether they were able to eat all food textures, and able to eat a meal or snack without drinking, were recorded word for word.

Diet quality was evaluated retrospectively by comparing dietary intake to the Mediterranean Diet Quality Index (KIDMED) (28). The index consists of 16 questions: 12 with a positive connotation (score of +1) and four with a negative connotation (score of −1) in relation to the Mediterranean diet (MD). Scores ≥8 are considered “optimal MD,” scores 4–7 as “intermediate diet quality” and scores ≤3 as “very low diet quality.” To account for one missing question (“use of olive oil,” due to uncertain data) in the dietary interview, one point was added to the total score in all patients.

### 2.7. Statistical analyses

The data were analyzed using Stata Statistical Software, version 17 (StataCorp LLC). The results are presented as mean ± standard deviation (SD), and median with interquartile range (IR). The non-parametric Wilcoxon Rank Sum Test was used for the intergroup comparisons. Spearman’s rho was applied for correlations between variables, and the chi-square test and Fisher exact test was used for binary outcomes. Statistical values were considered significant when *p* < 0.05. Mean values were used for missing and “*I do not know*” answers.

## 3. Results

### 3.1. Demographic data

Twenty-three patients (mean age 36 years) were recruited during elective short-term hospitalization in psychiatric ward. The demographic data and characteristics of patients and age and gender-matched controls are presented in Table 1. The groups were comparable concerning age, gender, ethnicity, education level and BMI, however the patient group had a lower employment level, a higher prevalence of current smokers and they used more medications.

**Table 1:** Characteristics of the patients and healthy controls.

	Patients (*n* = 23)	Controls (*n* = 23)	value of *p*
**Age (year)**			
Mean ± SD	36.2 ± 13.2	42.0 ± 17.6	0.435
Median (IR)	33 (24–50)	44 (22–57)	
**Gender**			
Female *n* (%)	15 (65%)	15 (65%)	1.000
Male *n* (%)	8 (35%)	8 (35%)	
**Ethnicity**			
Scandinavian	17 (74%)	18 (78%)	0.384
European	4 (17%)	2 (9%)	
Other	2 (8%)	3 (12%)	
**Education level**			
Basic (7–10 years)	3 (13%)	0	0.056
Secondary (10–13 years)	4 (17%)	1 (4%)	
Higher (>13 years)	16 (70%)	22 (96%)	
**Occupation**			
Working	2 (9%)	14 (61%)	<0.001*
Sick leave/rehabilitation/disabled	13 (57%)	1 (4%)	
Unemployed	6 (26%)	0	
Student	2 (9%)	8 (35%)	
**Smoking status**			
Current smoker	9 (39%)	0	0.001*
**Number of medications**			
Mean ± SD	5.0 ± 2.4	1 ± 0	<0.001*
Median (IR)	4 (3–7)	1 (1–1)	
**BMI**			
Mean ± SD	25.1 ± 5.6	22.8 ± 2.6	0.247
Median (IR)	23.5 (22–29)	(20–25)	

Values are presented as mean ± SD, range, number of cases (percentage), and median (interquartile range, IR) *p < 0.05.

The patients took on average five medications daily, most commonly antidepressants, antipsychotics, or a combination of these, as presented in Table 2. Detailed information on all medications can be found in Supplementary Table S1. On average, three of these medications had xerostomia as a known side effect. The most common patient diagnoses were personality disorders, depression, anxiety, post-traumatic stress disorder, anorexia nervosa, disturbance of activity and attention disorders, psychotic disorders and bipolar disorders, and 61% had more than one of these psychiatric diagnoses, as seen in Table 2. Five patients were also diagnosed with eating disorders, and another four gave unsolicited information about struggling with a disordered eating pattern in the past.

**Table 2:** Frequency of the most common psychiatric diagnoses and prescribed psychiatric medications for the patients, values shown as number of patients (percent).

	Patients *n* (%)
**Psychiatric diagnoses**	
Personality disorder	8 (35)
Depression	8 (35)
Anxiety	6 (26)
Post-traumatic stress disorder	5 (22)
Anorexia Nervosa (including atypical anorexia nervosa)	5 (22)
Disturbance of activity and attention (including ADHD)	5 (22)
Psychotic disorders	4 (17)
Bipolar disorders	3 (13)
More than one of the above diagnoses	14 (61)
**Prescribed psychiatric medications**	
Only antidepressants	8 (35)
Antidepressants and antipsychotic drugs	6 (27)
Only antipsychotic drugs	4 (17)
Antipsychotic drugs, anxiety drugs, and hypnotic drugs	1 (4)
Antidepressants, antipsychotic drugs, and anxiety drugs	1 (4)
Antipsychotic drugs and sedative/hypnotic drugs	1 (4)
Antidepressants, antipsychotic drugs and sedative/hypnotic drugs	1 (4)
Antidepressants and sedative/hypnotic drugs	1 (4)

### 3.2. Oral parameters

The patients reported significantly poorer oral and general health compared to the controls, as shown in Table 3. The UWS secretion rate was within the normal range and not significantly different from controls, although 26% of the patients had hyposalivation with respect to UWS secretion. The SWS secretion rate was significantly lower in the patients than in the controls (1.41 ± 0.76 vs. 2.0 ± 0.8, *p* < 0.05), and four patients (17%) had hyposalivation with respect to SWS secretion. The same four patients had hyposalivation in the unstimulated state. The patient group demonstrated more clinical findings of oral dryness than the controls (CODS: 5.1 ± 1.9 vs. 0.6 ± 1.2, *p* < 0.001), and seven (30%) of the patients had severe oral dryness findings with a CODS >6. The results of the salivary secretion measurements and the clinical oral dryness findings are shown in Table 4.

**Table 3:** Results from the self-reported rating of oral and general health on a scale from 0 to 4, 0 = very bad and 4 = very good.

	Patients (*n* = 23)	Controls (*n* = 23)	value of *p*
**Self-reported oral health (score range 0–4)**			
Mean ± SD	2.1 ± 1.1	3.4 ± 0.6	<0.001*
Median (IR)	2 (1–3)	3 (3–4)	
**Self-reported general health (score range 0–4)**			
Mean ± SD	1.8 ± 0.9	3.8 ± 0.4	<0.001*
Median (IR)	2 (1–3)	4 (4–4)	

Values are presented as mean ± SD, and median (interquartile range, IR). *p < 0.05.

**Table 4:** Results from unstimulated whole saliva (UWS) secretion, stimulated whole saliva (SWS) secretion, CODS (Clinical Oral Dryness Score), and dental status.

	Patients (*n* = 23)	Controls (*n* = 23)	value of *p*
**UWS ml/min**			
Mean ± SD	0.30 ± 0.17	0.4 ± 0.2	0.072
Median (IR)	0.29 (0.12–0.41)		
>0.1 and <0.3 *n* (%)	7 (30%)	6 (26%)	1.000
≤0.1 *n* (%)	6 (26%)	1 (4%)	0.096
**SWS ml/min**			
Mean ± SD	1.41 ± 0.76	2.0 ± 0.8	0.012*
Median (IR)	1.4 (0.9–1.82)		
>0.7 and <1.5 *n* (%)	13 (57%)	7 (30%)	0.136
≤0.7 *n* (%)	4 (17%)	0 (0%)	0.109
**CODS (score range 0–10)**			
Mean ± SD	5.1 ± 1.9	0.6 ± 1.2	< 0.001*
Median (IR)	5 (4–7)	0 (0–1)	
CODS >6 *n* (%)	7 (30%)	0 (0%)	
**Total number of teeth present (score range 0–28)**			
Mean ± SD	25.3 ± 4.9	27 ± 1.7	0.266
Median (IR)	28 (6–28)	28 (23–28)	
**Number of decayed teeth (score range 0–28)**			
Mean ± SD	0.5 ± 1.1	0.2 ± 0.6	0.486
Median (IR)	0 (0–2)	0 (0–2)	
**Number of filled teeth (score range 0–28)**			
Mean ± SD	5.4 ± 5.5	7.9 ± 6.1	0.155
Median (IR)	5 (0–18)	6 (0–20)	
**Caries experience (score range 0–28)**			
Mean ± SD	8.5 ± 8.1	8.6 ± 6.5	0.582
Median (IR)	5 (0–28)	7 (1–20)	

Values are presented as mean ± SD, median (interquartile range, IR), and number of cases (percentage). *p < 0.05.

Xerostomia was more pronounced in the patient group compared to the control group, SXI scores were significantly higher in the patients (10.4 ± 2.7 vs. 6 ± 0.7, *p* < 0.001), and the most frequent complaints were *“my lips feel dry”* and *“my mouth feels dry,”* as shown in Table 5.

**Table 5:** Shortened xerostomia inventory (SXI) scores and frequency of complaints for each question, presented as mean ± SD, median (interquartile range, IR), and number of cases (percentage).

	Patients (*n* = 23)	Controls (*n* = 23)	value of *p*
**SXI (score range 5–15)**			
Mean ± SD	10.4 ± 2.7	6 ± 0.7	<0.001*
Median (IR)	11 (9–12)	6 (5.5–6.5)	
**SXI questions^a^**	**Never**	**Sometimes**	**Often**
My mouth feels dry when eating a meal	5 (22%)	11 (48%)	7 (30%)
My mouth feels dry	3 (13%)	10 (43%)	10 (43%)
I have difficulties eating dry foods	10 (43%)	5 (22%)	8 (35%)
I have difficulties swallowing certain foods	12 (52%)	6 (26%)	5 (22%)
My lips feel dry	3 (13%)	8 (35%)	12 (52%)

^a^: patients only. *p < 0.05.

### 3.3. Dental status

The overall dental status was similar in patients and controls, as shown in Table 4. Both groups had a median of 28 teeth, and the caries experience (D_3_MFT) in the patient group did not differ significantly from the control group (8.5 ± 8.1 vs. 8.6 ± 6.5, *p* = 0.582, respectively). Both groups had an average of less than one decayed tooth, and six participants in both groups (26%) had at least one root-filled tooth. Although a greater proportion of patients than controls had signs of dental wear (56% vs. 35%, *p* = 0.236), and visible dental biofilm (30% vs. 13%, *p* = 0.284), these differences were not statistically significant. Significantly more patients had signs of gingivitis than controls (31% vs. 0%, *p* < 0.05), and among the patients, 29% had either localized or generalized periodontitis.

### 3.4. Dental visits and oral hygiene routines

Seventy-eight percent of controls and 57% of patients had visited the dentist or dental hygienist in the last 24 months. Among the patients who had visited the dentist/dental hygienist in the last 24 months, 75% went for a regular dental check-up, two patients (17%) had attended for treatment of pain, and one patient (8%) had received planned treatment. Among the patients who had not visited a dentist/dental hygienist in the last 24 months, three (33%) could not afford it, two (22%) were too afraid to make a visit, and two (22%) did not feel the need to visit the dentist.

Regarding oral hygiene routines, 13 patients (62%) reported brushing their teeth twice a day, 7 patients (33%) brushed daily, and one patient reported brushing about once a week. Most patients (81%) seldom or never performed interdental cleaning. Dental floss was the most common interdental cleaning method, followed by interdental brushes and toothpicks.

### 3.5. Smell and taste function

The patients reported lower smell scores compared to the controls (6.9 ± 2.1 vs. 8.3 ± 1.8, *p* < 0.05). However, objective measurements were comparable to controls (9.9 ± 1.7 vs. 10.1 ± 1.6, *p* = 0.096), as shown in Table 6. There were no statistically significant differences in the smell function classifications, as the prevalence of anosmic, hyposmic and normosmic patients were comparable to the controls.

**Table 6:** Results and classifications of smell function measurements.

	Patients (*n* = 23)	Controls (*n* = 23)	Value of *p*
**Self-reported smell function (score range 1–10)**			
Mean ± SD	6.9 ± 2.1	8.3 ± 1.8	0.017*
Median (IR)	7 (6–8)	9 (7–10)	
**Measured smell function (score range 0–12)**			
Mean ± SD	9.9 ± 1.7	10.1 ± 1.6	0.962
Median (IR)	10 (9–11)	10 (9–11)	
**Smell function classification (score range)**			0.580
Anosmic (0–5) *n* (%)	0 (0)	1 (4)	
Hyposmic (6–9) *n* (%)	6 (26)	5 (22)	
Normosmic (10–12) *n* (%)	17 (74)	17 (74)	

Result values represent mean ± SD and median (interquartile range, IR) of self-reported visual analog scale scores and objectively measured scores with Sniffin’ sticks. Smell function classification of the patients and controls are categorized according to the olfactory classification as number of cases (percentage). *p < 0.05.

The same tendency was seen for taste function, as the self-reported taste scores were lower in the patient group compared to the controls (6.9 ± 2.2 vs. 8.6 ± 1.6, *p* < 0.05), but comparable when measured objectively (11.8 ± 3.2 vs. 12.2 ± 2.0, *p* = 0.987). Classification of taste function showed similar results for ageusic, hypogeusic and normogeusic for patients and controls, as shown in Table 7.

**Table 7:** Results and classification of taste function measurements.

	Patients (*n* = 23)	Controls (*n* = 23)	Value of *p*
**Self-reported taste function (score range 1–10)**			
Mean ± SD	6.9 ± 2.2	8.6 ± 1.6	0.004*
Median (IR)	7 (6–8.5)	9 (8–10)	
**Measured taste function (score range 0–16)**			
Mean ± SD	11.8 ± 3.2	12.2 ± 2.0	0.987
Median (IR)	12 (11–14)	13 (11–13)	
**Taste function classification (score range)**			0.297
Ageusic (0) *n* (%)	0 (0)	0 (0)	
Hypogeusic (1–8) *n* (%)	4 (17)	2 (9)	
Normogeusic (9–16) *n* (%)	19 (83)	21 (91)	

Result values represent mean ± SD and median (interquartile range, IR) of self-reported visual analog scale scores and objectively measured scores with taste strips. Taste function classification of the patients and controls are categorized according to the gustatory classification as number of cases (percentage). *p < 0.05.

Complaints of halitosis and dysgeusia were significantly more frequent in the patient group than in the control group (48% vs. 4%, *p* < 0.05, and 43% vs. 4%, *p* < 0.05, respectively). Previous candida infection was more common for the patients, but did not reflect current candida growth, as presented in Table 8.

**Table 8:** Frequency of complaints about halitosis, burning mouth sensation and dysgeusia, and previous and current oral candida infection.

	Patients (*n* = 23)	Controls (*n* = 23)	Value of *p*
**Oral disorders**			
Halitosis	10 (48%)	1 (4%)	0.001*
Burning mouth sensation	2 (10%)	1 (4%)	0.599
Dysgeusia	9 (43%)	1 (4%)	0.003*
**Candida**			
Previous candida infection	6 (26%)	0 (0%)	0.022*
Current candida growth	0.6 ± 0.7	0.4 ± 0.7	0.311

Current oral candida infection was scored semi-quantitatively (0 = no growth, 1 = minimal growth, 2 = moderate growth, 3 = severe growth). Values are presented as number of cases (percentage) and as mean ± SD. *p < 0.05.

### 3.6. Oral health-related quality of life

The patient group had significantly higher OHIP-14 scores compared to the control group (17.9 ± 12.7 vs. 1.04 ± 1.9, *p* < 0.001), indicating a poorer oral health-related quality of life. Table 9 shows that “psychological discomfort,” “psychological disability” and “physical pain” were the OHIP-14 dimensions with the highest frequency of complaints (43, 39 and 39%, respectively).

**Table 9:** The Oral Health Impact Profile-14 total score (OHIP-14), dimension scores and frequency of patients answering either “fairly often” or “very often” on the specific OHIP-14 dimensions (shown in bold) and the corresponding questions (Q).

	Patients (*n* = 23)	Controls (*n* = 23)
**Total OHIP-14 (score range 0–56)**		
Mean ± SD	17.9 ± 12.7	1.04 ± 1.9^b^
Median (IR)	16 (8–28)	0 (0–1)
**Dimensions (patients only)**	**Number (%) of patients answering “*fairly often*” or “*very often*” (%)**	**Mean ± SD**
**Functional limitations (Q1 + Q2)**	**2 (9)**	1.2 ± 1.8
Trouble pronouncing words	2 (9)	
Worsened taste	1 (4)	
**Physical pain (Q3 + Q4)**	**9 (39)**	3.3 ± 2.3
Aching in the mouth	7 (30)	
Discomfort eating food	4 (17)	
**Psychological discomfort (Q5 + Q6)**	**10 (43)**	4.5 ± 2.8
Feeling self-conscious	10 (43)	
Feeling tense	7 (30)	
**Physical disability (Q7 + Q8)**	**3 (13)**	1.6 ± 2.3
Poor diet	3 (13)	
Interrupted meals	3 (13)	
**Psychological disability (Q9 + Q10)**	**9 (39)**	3.6 ± 2.4
Difficulties relaxing	6 (26)	
Embarrassment	7 (30)	
**Social disability (Q11 + Q12)**	**1 (4)**	1.3 ± 1.7
Irritability of other people	0 (0)	
Difficulties doing usual jobs	1 (4)	
**Handicap (Q13 + Q14)**	**5 (22)**	2.1 ± 2.3
Life less satisfying	4 (17)	
Inability to function	2 (9)	

Values are presented as mean ± SD, median (interquartile range, IR), and number of cases (percentage). *p < 0.05.

^b^: *p* < 0.001.

Dry mouth and halitosis were the most frequent oral disorders stated by the patients to reduce their social life, followed by “reduced smell function,” “bad taste in mouth,” “reduced taste function” and “stinging or burning sensation in the mouth,” as seen in Table 10.

**Table 10:** Number (percentage) of patients self-reporting reduced social life due to oral disorders.

Self-reported reduced social life due to	*n* (%)
Dry mouth	9 (39)
Halitosis	6 (26)
Reduced smell function	5 (22)
Bad taste in the mouth	5 (22)
Reduced taste function	4 (17)
Stinging or burning sensation in the mouth	2 (9)

### 3.7. Nutritional status and risk assessment

The nutritional screening using the PG-SGA-SF showed that four patients (18%) were at high risk (score ≥9) of malnutrition, ten (46%) were at medium risk (score 4–8), and eight (36%) were at low risk (score 0–3). Table 11 presents the results from the different components from PG-SGA-SF and the sum score.

**Table 11:** Component scores and sum score for the Patient-Generated Subjective Global Assessment Short Form (PG-SGA-SF).

Malnutrition risk (score range)	*n* (%)	
High risk (score ≥9)	4 (18)	
Medium risk (score 4–8)	10 (46)	
Low risk (score 0–3)	8 (36)	
**PG-SGA-SF components (score range)**	**Mean ± SD**	**Median (IR)**
Weight changes (0–5)	0.4 ± 0.8	0 (0–0)
Food intake (0–4)	0.4 ± 0.5	0 (0–1)
Symptoms (0–24)	3.1 ± 2.9	3.5 (0–4)
Activity (0–3)	1.4 ± 1.0	1 (1–2)
PG-SGA-SF (0–36)	5.2 ± 3.4	5.5 (2–7)

Values shown as number of cases (percentage), mean ± SD and median (interquartile range, IR). Higher score indicates more severe nutritional problems.

Of the four patients at high risk of malnutrition, two had normal weight (BMI 18.5 to <25) and two were in the obese range (BMI ≥30). Of those at medium risk of malnutrition, one was underweight (BMI <18.5), four had normal weight and five were in the overweight/obese range (BMI ≥25). The group at low risk of malnutrition included three underweight patients with eating disorders.

Activity and function levels were low for many of the patients. Three (14%) reported to spend most of their day in bed or in a chair, and only four (18%) described their activity level as normal with no limitations. The symptoms component of PG-SGA-SF contributed the most to high total scores. Of the four high-risk patients, the total score was due to the symptom component alone in one patient, and due to the combination of the symptom and activity component in the other three patients. Of the ten patients at medium risk, the score was due to the symptom component alone in seven patients. Those in the low-risk group reported few or no symptoms.

**Figure 1:**
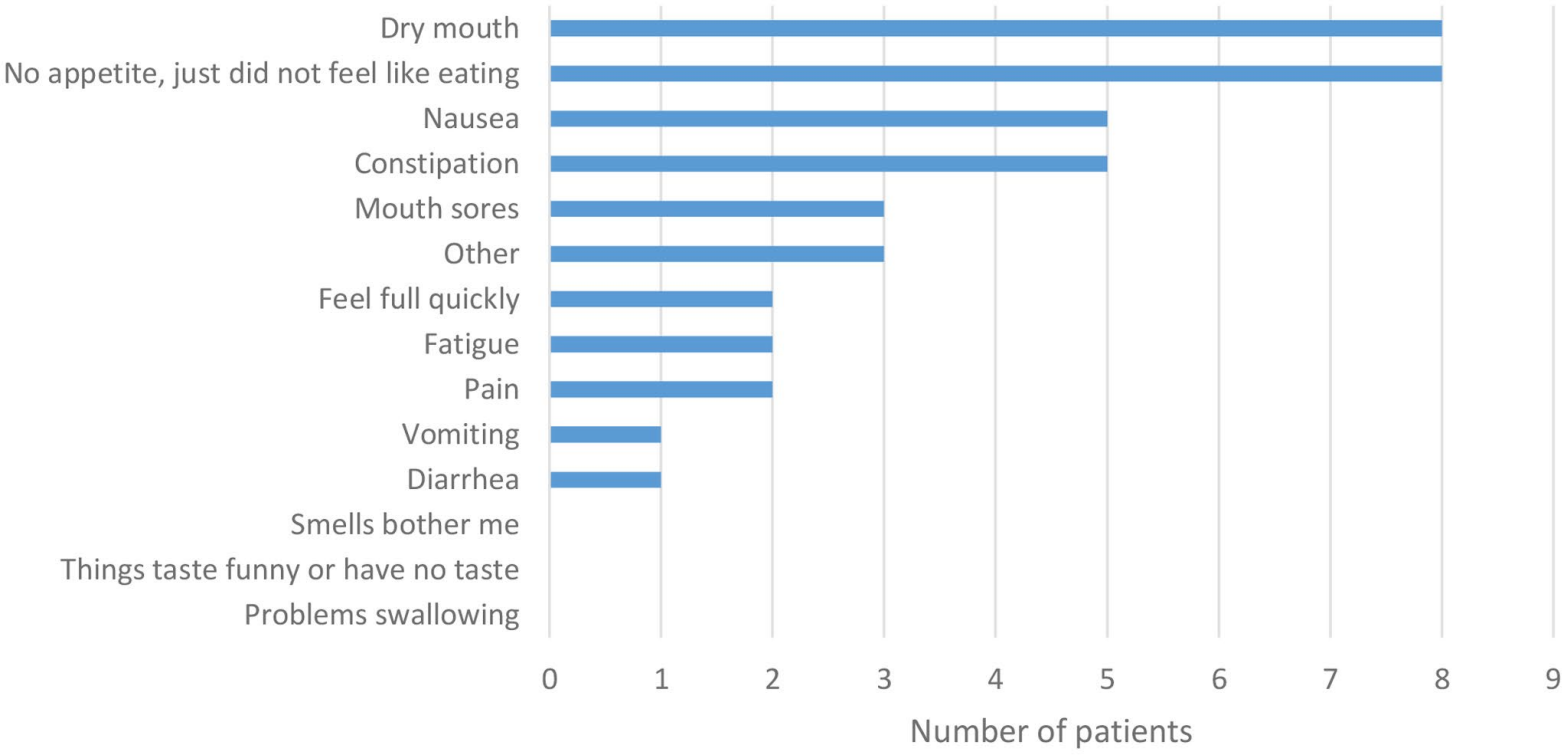
The effect of various symptoms on food intake. The X-axis indicates the number of patients that had recorded specific symptoms in reply to the Patient-Generated Subjective Global Assessment Short Form question (PG-SGA-SF). “*I have had the following problems that have kept me from eating enough during the past 2 weeks*” (*n* = 22).

*“Dry mouth”* and “*No appetite, just did not feel like eating*” were the most frequent symptoms stated by the patients to keep them from eating enough, followed by *“nausea”* and *“constipation,”* as seen in Figure 1. Of the eight patients who reported that dry mouth kept them from eating enough, seven were at high or medium risk of malnutrition. Taste or smell (“*Things taste funny or have no taste*,” “*Smells bother me*”) were not reported to keep patients from eating enough.

### 3.8. Food habits and diet quality

**Figure 2:**
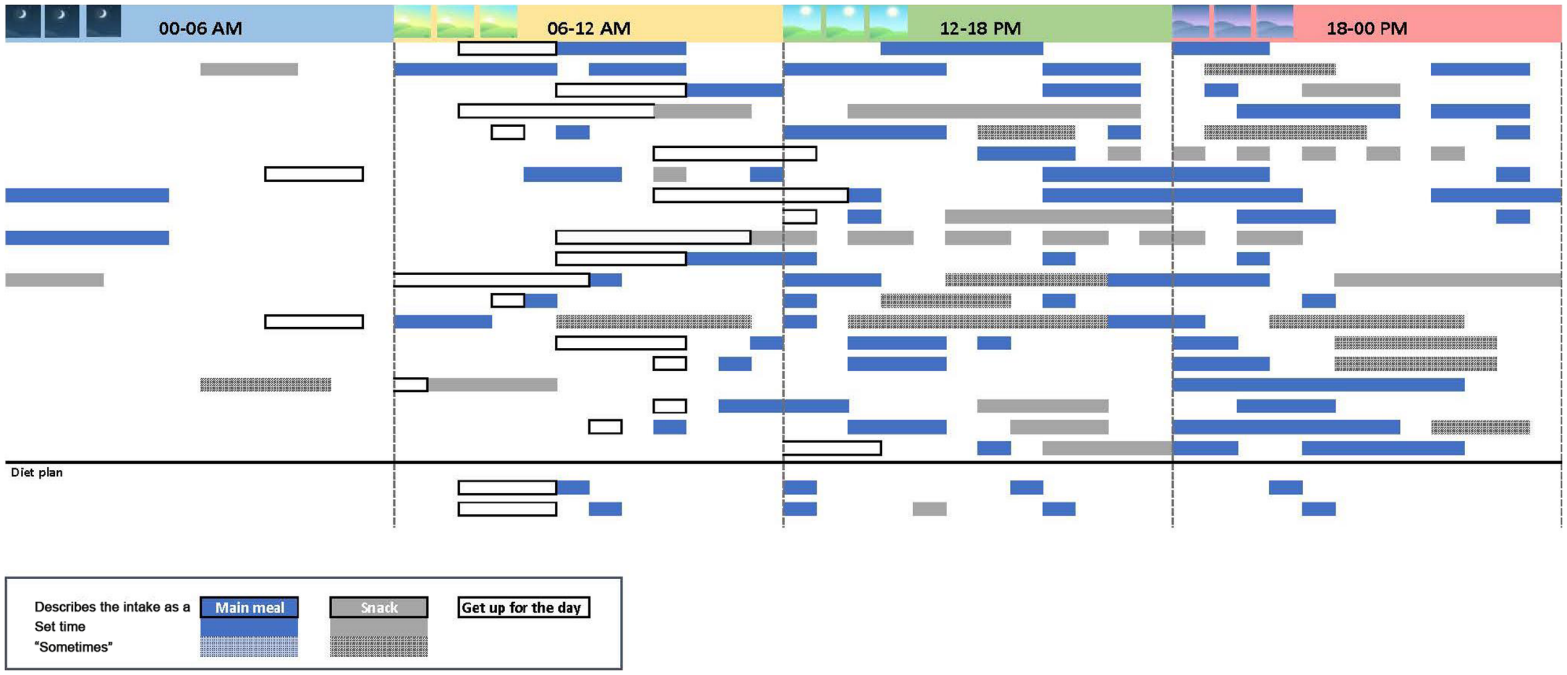
Illustration of individual meal patterns based on the dietary interviews. Each horizontal bar represent one patient. Long horizontal bars indicate time intervals for meals/snacks, rather than fixed hours. Two patients followed strict meal plans due to anorexia nervosa.

In general, the patient group reported irregular meal patterns, as shown in Figure 2. Many patients could only indicate periods of several hours because there were no exact mealtimes. In one patient, disturbed nocturnal habits due to compulsory habits made it impossible to report wake-up time. The first meal of the day was the easiest to define for most patients. Six patients did not eat breakfast, defined as a main meal eaten within 2 h after waking up and before noon. Five patients ate only one or two main meals during the day, while the rest ate at least three main meals a day. The number of snacks consumed during the day was very individual and difficult to define in time. Two patients regularly ate a main meal during the night, and for one of them, this was the only main meal.

**Figure 3:**
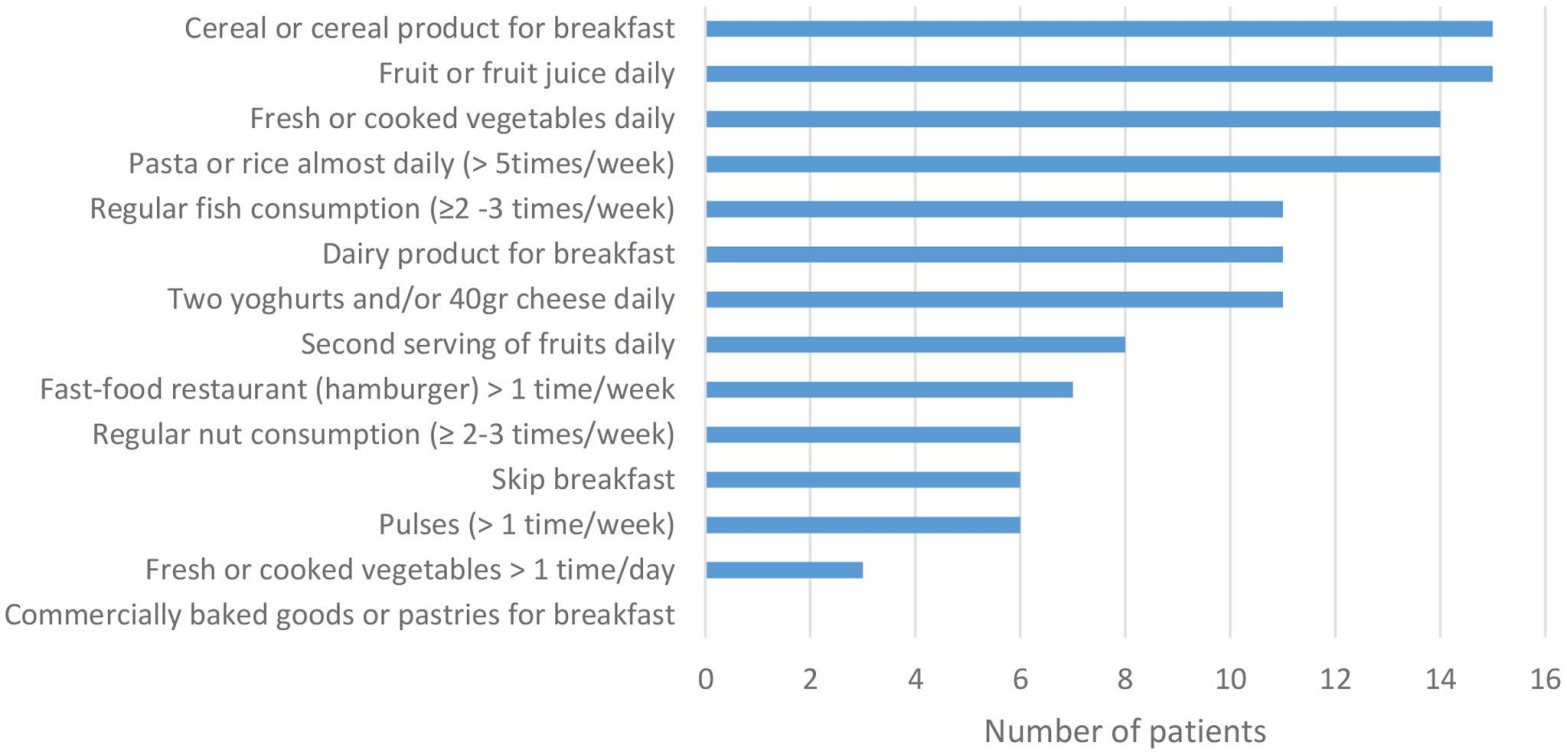
Number of patients with food habits corresponding to the separate index questions included in the Mediterranean Diet Quality Index (KIDMED), except for one missing question (use of olive oil). The numbers were based on the dietary interviews (*n* = 22).

The results from the KIDMED-index showed that diet quality was inadequate in 77% of the patients, five of the patients (23%) scored “a very low diet quality” (score ≤3) and 12 of the patients (55%) scored “intermediate diet quality” (score 4–7). The consumption of different food groups included in the index is shown in Figure 3. A second daily serving of vegetables or fruits, and weekly consumption of nuts and pulses (beans, lentils, and peas) were lowest at group level among food groups that contribute to a higher total index. Four patients (18%) did not eat any fruit or vegetables on a daily basis.

### 3.9. Relationships between oral findings and nutrition status

The PG-SGA-SF symptoms component score showed a strong negative correlation with the patients’ self-reported oral health evaluation (*r* = −0.717, *p* < 0.001), and a strong positive correlation with their oral health-related quality of life (OHIP-14) (*r* = 0.624, *p* = 0.002). There were no significant correlations between oral findings and diet quality. However, the dietary interviews gave some insight into how oral health problems, such as dry mouth, hypersensitive teeth and tooth decay affected the food habits in some patients. The following quotes give insight into compensating actions or avoidance of certain foods:

*Foods that are soft and not dry glide down more easily and are preferably chosen*.

*I can eat bread, but crispbread has a consistency that is too dry. Müsli needs a lot of yoghurt not to be too dry*.

Two patients explained how problems with teeth affected food choices:

*Hard vegetables and fruits can be difficult because my gums are frail/weak, and I have some fear that the teeth will be harmed*.

*I deliberately avoid hard fruits such as apple but can eat it grated. I rather choose soft fruit as banana. The same goes for raw vegetables - I avoid hard and raw ones because my teeth do not withstand them*.

The following quote illustrates the complexity of factors that may be present in this patient group:

*I feel that depression, economy, alcohol, sleepless nights and tooth problems make it difficult to eat enough*.

Table 12 shows a comparison of the results from several objective and subjective parameters in the patients (SXI, CODS, UWS, SWS, OHIP-14, PG-SGA-SF symptoms component score, number of drugs and number of drugs with dry mouth as a known side effect). The number of drugs with xerostomia as a side effect correlated with CODS, UWS, total number of medications and age (*r* = 0.512, *p* = 0.013, *r* = −0.457, *p* = 0.028, *r* = 0.725, *p* < 0.001, *r* = 0.625, *p* = 0.001, respectively). SXI showed no significant correlation with OHIP-14 or the symptoms component of PG-SGA-SF.

**Table 12:** Results from Summated Xerostomia Inventory (SXI), Clinical Oral Dryness Score (CODS), unstimulated whole saliva (UWS), stimulated whole saliva (SWS), Oral Health Impact Profile-14 (OHIP-14), the symptoms component of Patient-Generated Subjective Global Assessment Short Form (PG-SGA-SF), number of drugs and number of drugs with dry mouth as a known side effect for the specific patients.

Patient	Age (years)	SXI score	CODS	UWS (ml/min)	SWS (ml/min)	OHIP-14 score	PG-SGA-SF symptoms component score	Number of drugs	Number of drugs with dry mouth as known side effect
1	56	14	7	0.1	0.4	8	1	9	5
2	61	9	7	0.5	1.4	36	4	5	3
3	24	10	7	0.1	0.4	18	4	5	2
4	43	14	6	0.3	2.4	20	4	8	4
5	36	7	2	0.2	1.4	10	4	4	2
6	58	6	4	0.5	2.4	5	1	7	3
7	38	9	6	0.1	0.9	28	6	8	6
8	24	7	5	0.5	1.4	11	0	4	2
9	52	13	8	0.0	0.1	37	2	11	7
10	27	12	5	0.2	2.9	9	3	7	5
11	22	7	2	0.4	1.5	16	5	5	2
12	42	13	2	0.4	0.9	37	7	2	2
13	22	12	3	0.4	1.0	16	4	3	2
14	23	10	4	0.6	1.5	47	3	3	2
15	50	9	8	0.3	1.8	19	1	5	4
16	54	11	5	0.1	0.9	2	0	4	4
17	36	11	4	0.4	1.0	26	11	3	2
18	21	5	5	0.5	1.4	12	0	7	1
19	27	15	7	0.3	1.5	30	7	3	2
20	27	11	7	0.1	0.7	8	4	3	2
21	28	12	3	0.2	2.4	8	0	3	1
22	29	12	4	0.3	1.4	9	0	2	0
23	33	10	6	0.4	2.7	0	0	3	2
Mean	36	10	5	0.3	1.4	18	3	5	3

Values higher than mean (lower than mean for UWS and SWS) are marked in gray.

## 4. Discussion

### 4.1. Oral health and xerostomia

In this explorative study on a small group of patients in short-term psychiatric ward, the main oral findings were a high degree of xerostomia and poor oral health-related quality of life, and the main nutritional findings were irregular meal patterns and low diet quality. There was a strong positive correlation between oral health-related quality of life measured by OHIP-14, and the symptoms component score of the PG-SGA-SF nutritional screening tool.

Xerostomia seemed to be more pronounced in the patients than hyposalivation, as only six patients (23%) had pathologically reduced unstimulated saliva secretion, while 43% of the patients reported severe problems related to dry mouth (SXI answer *often* for *“my mouth feels dry”*). Dry mouth is a known side effect of many of the drugs used in the treatment of psychiatric patients. However, information provided by the industry about the side-effect “dry mouth” for different medications unfortunately does not distinguish between xerostomia and hyposalivation. In addition, the two terms are rarely separated when searching the literature for prevalence of hyposalivation (29).

Problems with xerostomia were revealed by several of the assessment tools, namely some questions of SXI (“*My mouth feels dry when eating a meal*” 78% and “*I have difficulties eating dry foods*” 57%), questions concerning impact on social life (“reduced social life due to dry mouth” 39%) and questions of the symptoms-component of PG-SGA-SF (*“dry mouth”* 36%). On the other hand, the responses from the various screening tools differed concerning other symptoms that potentially might affect food intake, such as difficulties with swallowing and bad taste. While 48% reported that *“I have difficulties swallowing certain foods”* from the SXI, no patients stated in the PG-SGA-SF that *“problems swallowing”* was a symptom that kept them from eating enough. Likewise, 43% of the patients complained of dysgeusia, but none stated in the PG-SGA-SF that *“things taste funny or have no taste”* kept them from eating enough. These examples illustrate that whether a question relates to the presence of a symptom or the consequence of a symptom (i.e., “*kept me from eating enough”*), may have different implications for actions. Of note, misunderstandings regarding completing the self-administered questionnaires may occur because the patient does not understand or misinterprets a question (30).

The patients were also troubled by halitosis and dysgeusia, and dry mouth and halitosis were the most frequent oral disorders stated by the patients that negatively affected their social life.

### 4.2. Dental health and habits

In contrast to previous studies where dental status was found to be poorer in patients with mental illness than in the general population (5, 8), caries experience (D_3_MFT) was similar in patients and controls in this study, and both groups had on average less than one decayed tooth. This finding may reflect the relatively good overall oral- and dental health in Norway. However, the small sample size in this study, relatively low mean age in the patient group, and the diversity of diagnoses, make comparisons with other studies difficult.

Interestingly, two systematic reviews found differences in the oral health status between patients with severe mental illness (defined as primary diagnosis of dementia, schizophrenia, bipolar affective disorder or other affective disorders) and those with more common psychological disorders (defined as primary diagnosis of depression, generalized anxiety disorder, panic disorder, obsessive–compulsive disorder, post-traumatic stress disorder and phobias) (9, 31). Patients had more caries experience than controls, however the difference was greater for those with more severe psychiatric illness, and this was suggested to be the result of poor oral hygiene and subsequent plaque formation and gingivitis (31).

Psychiatric patients have potentially many risk factors for developing poor dental health. The majority of patients in the present study reported brushing their teeth either twice a day (62%) or daily (33%), however, they seldom or never performed interdental cleaning. The lack of interdental cleaning by the patients in the present study was clearly reflected in their gingival status and was in line with previous studies showing that significantly more patients than controls have signs of gingivitis (5, 8). Another study reported that only one third of outpatients on psychiatric medications had visited a dentist in the previous two years (11). This is in contrast with the results in the present study, where 57% of the patients had visited the dentist or dental hygienist in the past two years. Within the publicly funded dental care in Norway, psychiatric patients can qualify for treatment on a voluntary basis. However, a third of the patients in this study were not aware of this possibility.

### 4.3. Oral health and diet

Irregular meal patterns were typical in the patients in the present study. This was expressed as few regular meals and frequent intake of snacks, which may in turn have a negative impact on general health, including oral health (32). Of note, irregular meal patterns were related to irregular sleeping patterns and irregular daily/nightly rhythms, as shown in Figure 2. This fact may partly explain these patients’ difficulties in adjusting to normal requirements of daily life. Low diet quality in the present study was partly due to low intake of vegetables, fruits, nuts, and pulses. These food groups have independently been demonstrated to inversely affect mental health (33). Hence, it is relevant to examine whether oral health problems are barriers to the consumption of healthy foods.

Psychiatric patients receiving dental treatment, have described that better masticatory functions improved their diet (34). In the present study, comments from patients with poor dental status indicated that problems with chewing hard foods might be one reason why they avoid healthy food groups such as vegetables and fruits. Oral dryness has been reported to lead to frequent intake of carbohydrate-rich foods, especially in combination with sugar cravings (34). These findings support the need to evaluate meal patterns and diet quality to prevent general health problems and deterioration of oral health in these patients.

As previously described, the patients were recruited from an elective psychiatric ward and had various diagnoses. Although the inclusion criteria were anxiety, psychosis and/or depression, many of the patients had several additional diagnoses, including eating disorders characterized by low body weight and food restriction in five patients. To be included in the study, BMI had to be ≥16, thus excluding the more severe cases of anorexia nervosa. However, low body weight can also occur among psychiatric patients due to other reasons such as depression (35), or drug abuse (36), although this was not the case among these patients.

As the PG-SGA-SF risk-screening tool assesses recent weight loss and not BMI, the low BMI of the patients with eating disorders did not affect the results of the nutrition risk screening. Regarding meal patterns, only two patients followed a diet plan prior to the hospital stay, whereas the others had a more irregular pattern like most of the other patients. The dietary intake may have been affected by the nature of the eating disorder, by low amounts of energy and nutrients, but the dietary quality was not affected in our study, this is in accordance with previous studies (37). In summary, including patients with eating disorders in the present study did not seem to affect the dietary results.

### 4.4. Clinical implications

The physical health of people with mental disorders is commonly overlooked by health care providers (38). According to Norwegian national guidelines for the prevention and treatment of malnutrition, all patients should be assessed for malnutrition upon admittance to health institutions (39). However, appropriate nutrition risk-screening tools targeted to mental health services independent of BMI, are lacking (40). The PG-SGA-SF risk-screening tool used in the present study is independent of BMI. It showed how the symptom component score regardless of weight loss and BMI indicated a high risk of malnutrition among the patients. In addition, the symptoms component score of PG-SGA-SF better reflected the patients’ oral health-related quality of life (OHIP-14 score) than the shortened xerostomia inventory (SXI). The PG-SGA-SF may therefore be a useful screening tool for both malnutrition risk, and oral-health related quality of life in these patients in a clinical setting.

Psychiatric health disorders are complex. Contributing factors include life-style factors such as diet, alcohol and drug use, stress, and socioeconomic status (41). In this setting, the results of oral and nutritional screenings must address the whole range of factors that can influence physical and psychological health. This was brilliantly pinpointed by one of the patients: *“I feel that depression, economy, alcohol, sleepless nights and tooth problems make it difficult to eat enough.”*

To our knowledge, this is the first exploratory study where both an extensive oral examination and a nutritional assessment have been performed on an albeit limited number of patients in short-term psychiatric ward. A strength of the study is that all eligible participants were requested to take part in the study, and that the examinations were performed according to a standardized predefined protocol. However, two obvious limitations of the study were the diversity in psychiatric diagnoses and the low number of patients. The extensive, rather time-consuming protocols were carefully carried out in a small number of these vulnerable patients to provide a basis for future studies. This approach was chosen to detect major differences between the patients and control participants. However, further research is needed to better understand the oral- and nutritional challenges of medicated psychiatric patients.

## 5. Conclusion

This limited group of patients in short-term psychiatric ward had both reduced oral health and a poor oral health-related quality of life. Furthermore, the patients’ nutritional intake was negatively affected by their oral health problems. Larger groups need to be studied; however, these findings suggest that oral health indicators and nutritional status of patients in need of psychiatric care are important for overall care and should be carefully evaluated and adjusted to prevent long-term health consequences.

## Data availability statement

The raw data supporting the conclusions of this article will be made available by the authors, without undue reservation.

## Ethics statement

The studies involving human participants were reviewed and approved by Norwegian Regional Medical Ethical Committee. The patients/participants provided their written informed consent to participate in this study.

## Author contributions

JF contributed to the clinical oral examinations and interviews of the patients, manuscript drafting, manuscript writing and revision. HH contributed to planning and carrying out the collaboration with Lovisenberg Diaconal Hospital, contributed to the Ethical committee application, contributed to the clinical oral examinations and interviews of patients and controls, manuscript drafting, manuscript writing and revision. KR contributed to the nutritional interviews, drafting the manuscript, manuscript writing and revision. TM-O contributed to planning the nutritional interviews, drafting the manuscript, manuscript writing and revision. AY, LH, and MR contributed to the clinical dental examinations and interviews of patients and controls, manuscript writing, editing and revision. PS contributed to the smell and taste evaluation of patients and controls, manuscript writing and revision. AH contributed with recruitment of patients, collaboration with Lovisenberg Diaconal hospital and psychiatric information and insight, manuscript writing and revision. JLJ was the principal investigator and supervisor, planned the study, provided funding from Faculty of Dentistry, planned, and collaborated with Lovisenberg Diaconal Hospital, wrote the Ethical committee application, contributed to the clinical oral examinations and interviews of patients and controls, manuscript drafting, manuscript writing and revision. All authors contributed to the article and approved the submitted version.

## Funding

The study was funded by the Faculty of Dentistry, University of Oslo, Norway.

## Conflict of interest

The authors declare that the research was conducted in the absence of any commercial or financial relationships that could be construed as a potential conflict of interest.

## Publisher’s note

All claims expressed in this article are solely those of the authors and do not necessarily represent those of their affiliated organizations, or those of the publisher, the editors and the reviewers. Any product that may be evaluated in this article, or claim that may be made by its manufacturer, is not guaranteed or endorsed by the publisher.
