# Supplementary material for: Exploring oral health indicators, oral health-related quality of life and nutritional aspects in 23 medicated patients from a short-term psychiatric ward
Source: Front Public Health. 2023 Apr 12;11:1083256. doi: 10.3389/fpubh.2023.1083256 (PMC10130439; doi:10.3389/fpubh.2023.1083256)
Supplement: Supplementary file 1 [file Table_1.pdf]

ATC classification		Number of patients
**Antipsychotics**		
N05A H04	Quetiapine	10
N05A H03	Olanzapine	3
N05A X12	Aripiprazole	2
N05A X08	Risperidone	1
N05A F03	Chlorprothixene	1
N05A E04	Ziprasidone	1
N05A N01	Lithium	1
**Antidepressants**		
**TeCAs**		
N06A X11	Mirtazapine	4
N06A X03	Mianserin	1
**SMSs**		
N06A X26	Vortioxetine	3
**MAOIs**		
N06A G02	Moclobemide	1
**SNRIs**		
N06A X16	Venlafaxine	4
N06A X21	Duloxetine	1
N06B A09	Atomoxetine	1
**Antiepileptics and bipolar treatment drugs**		
N03A X09	Lamotrigine	4
N03A G01	Valproic acid	1
**SSRIs**		
N06A B03	Fluoxetine	3
N06A B10	Escitalopram	3
N06A B06	Sertraline	1
**Sympathomimetic drugs**		
N06B A12	Lisdexamfetamine	2
N06B A02	Dextroamphetamine	1
**NDRI**		
N06B A04	Methylphenidate	1
**Anxiety drugs**		

N05B A04	Oxazepam	2
**Sedatives/hypnotics**		
N05C H01	Melatonin	3
N05C F01	Zopiclone	1
**Opioid antagonists**		
N07B B05	Nalmefene	1
**Stimulants of autonomic ganglia**		
N07B A01	Nicotine	1
**PPIs**		
A02B C05	Esomeprazole	3
A02B C01	Omeprazol	1
**Laxatives**		
A06A D65	Macrogol	3
**Antidiabetics**		
A10B A02	Metformin	1
A10B K01	Dapagliflozin	1
**Digestive enzymes**		
A09A A02	Lipase and Protease	1
**NSAIDs**		
B01A C06	Acetylsalicylic acid	1
**Agents acting on the renin- angiotensin system**		
C09A A02	Enalapril	1
C09C A06	Candesartan	1
**Lipid modifying agents**		
C10A A05	Atorvastatin	1
**Diuretic drugs**		
C03D A01	Spironolactone	1
**Conception control drugs**		
G03A A12	Drospirenon, Etinyløstradiol	1
**5α-reductase inhibitors**		
G04C B01	Finasteride	1
**Hormones**		
H03A A01	Levothyroxine	2
**Antiviral drugs**		
J05A R13	Abacavir, Dolutegravir, Lamivudine	1

**Bisphosphonate drugs**		
M05B A04	Alendronic acid	1
**Antihistamine drugs**		
R06A E07	Cetirizine	2
R06A X27	Desloratadine	2
S01G X02	Levocabastine	2
R01A D58	Azelastine + Fluticasone	2
R06A X13	Loratadine	1
**Drugs for obstructive airway diseases**		
R03A K07	Budesonide, Formoterol	1
R03A L08	Fluticasone furoate/ umeclidinium bromide/vilanterol	1
R03D C03	Montelukast	1
R03A L06	Olodaterol, Tiotropium bromide	1
R03A CO2	Salbutamol	1
R03B A05	Fluticasone	1

Table S1 Frequency of specified medications in the study population with Anatomical Therapeutic Chemical (ATC) classification and generic names. TeCAs, TetraCyclic Antidepressants; SMSs, Serotonin Modulators and Stimulators; MAOIs, MonoAmine Oxidase Inhibitors; SNRIs, Serotonin-Norepinephrine Reuptake Inhibitors; SSRIs, Selective Serotonin Reuptake Inhibitors; NDRI, Norepinephrine-Dopamine Reuptake Inhibitors; PPIs Proton Pump Inhibitors, NSAIDs NonSteroidal Anti-Inflammatory Drugs.
